# Deleterious variants in TAF7L cause human oligoasthenoteratozoospermia and its impairing histone to protamine exchange inducing reduced *in vitro* fertilization

**DOI:** 10.3389/fendo.2022.1099270

**Published:** 2023-01-11

**Authors:** Haowei Bai, Yanwei Sha, Yueqiu Tan, Peng Li, Yuxiang Zhang, Junwei Xu, Shuai Xu, Zhiyong Ji, Xiaobo Wang, Wei Chen, Jianxiong Zhang, Chencheng Yao, Zheng Li, Erlei Zhi

**Affiliations:** ^1^ Department of Andrology, Center for Men’s Health, Department of ART, Institute of Urologic Medical Center, Shanghai General Hospital, Shanghai Key Lab of Reproductive Medicine, Shanghai Jiao Tong University School of Medicine, Shanghai, China; ^2^ Department of Andrology, Women and Children’s Hospital, School of Medicine, Xiamen University, Xiamen, Fujian, China; ^3^ Institute of Reproductive and Stem Cell Engineering, School of Basic Medical Science, Central South University, Changsha, China

**Keywords:** *TAF7L*, hemizygous variant, oligoasthenoteratozoospermia, ICSI, male infertility

## Abstract

**Introduction:**

Oligoasthenoteratozoospermia (OAT) is a major cause of infertility in males. Only a few pathogenic genes of OAT have been clearly identified till now. A large number of OAT-affected cases remain largely unknown.

**Methods:**

Here, Whole-exome sequencing (WES) in 725 idiopathic OAT patients was performed. Ejaculated spermatozoa by OAT patients were microinjected into mouse oocytes to estimate fertilization potential. Diff-quick staining and transmission electron microscopy were performed to evaluate sperm morphology and ultrastructure. The protein expression level and localization In vitro were detected by Western Blotting and Immunocytochemistry.

**Results:**

We identified four X-linked hemizygous deleterious variants of TAF7L—namely, c.1301_1302del;(p.V434Afs*5), c.699G>T;(p.R233S), c.508delA; (p. T170fs), c.719dupA;(p.K240fs) —in five probands. Intracytoplasmic sperm injection (ICSI) were carried out in M1, M2-1and M3 patient's wife. However only M1 patient’s wife became pregnant after embryo transfer. In vitro study demonstrated significantly reduced fertilization ability in patient with TAF7L mutation. The TAF7L mutation let to abnormal sperm head and impaired histone-to protamine exchange. Variant 719dupA (p. K240fs) resulted in producing a truncated TAF7L protein and localized massively within the nucleus. In addition, TAF7L expression were not able to be detected due to variants c.1301_1302del (p. V434Afs*5) and c.508delA (p. T170fs) In vitro.

**Conclusion:**

Our findings support that TAF7L is one of pathogenic genes of OAT and deleterious mutations in TAF7L may cause impaired histone-to-protamine affected the chromatin compaction of sperm head.

## Introduction

Infertility affects about 15% of couples worldwide and a male infertility associated factor could be found in approximately half of all the couples (1, 2). Clinical manifestations of male infertility are mainly as follows, oligozoospermia, asthenozoospermia, teratozospermia, astheno-teratozospemia and oligoasthenoteratozoospermia(OAT) (3, 4). Varicocele, poor drug delivery, Y chromosomal microdeletion, and genetic abnormalities all contribute to male infertility (5).

Genetic research of male infertility related defect spermiogenesis has achieved some progress in recent years. Based on the above researches, different pathogenic genes have been classified as two specific phenotypes, including sperm head malformations (*AURKC, SPATA16*, *DPY19L2*, *PICK1*, *IQCN*), and multiple morphological abnormalities of the sperm flagella (*DNAH1*, *CFAP65*, *CFAP47, CFAP44*, *CFAP43*, *CFAP251, DZIP1, DNAH8, DNAH10*) *(* 3, 6–14). However, the discovery of different pathogenic genes indicates that there is great genetic heterogeneity in male infertility, and abnormal sperm with the same morphology may also be caused by different genes. Therefore, the discovery of more pathogenic genes has an important role in the genetic analysis of male infertility.

Sex chromosomes not only determine the sex of a baby but also play a vital part in fertility. According to previous research, most of the genes that are expressed predominantly in testes were identified on sex chromosomes (15). Deletion mutations in certain genes can cause male infertility (16). It is well known that microdeletions in the Y chromosome play important role in the infertility of males, however, only a few genes present on the X chromosome are known to cause male infertility. For instance, it has been found that the hemizygous *TEX11* variant causes meiosis arrest, giving rise to non-obstructive azoospermia (NOA) (17, 18). Also, hemizygous *ADGRG2* variants were identified in obstructive azoospermia (OA)-affected patients (19). Hemizygous *CFAP47* variants induced OAT and primary male infertility (12). However, many other X-linked genes responsible for male infertility related defect spermatogenesis are yet to be identified.

Another X-linked gene, *TAF7L* (also known as *CT40*), was linked to OAT and male infertility in the current study, and four X-linked hemizygous deleterious variations of *TAF7L* were detected in probands, but with significantly reduced *in vitro* fertilization. The results of this study will help in genetic counselling and treatments of infertility.

## Material and methods

### Study participants

In the current study, 725 idiopathic OAT patients were recruited. According to the WHO Laboratory Manual for the Examination and Processing of Human Semen (5th edition), OAT was defined as sperm concentration <15×10^6^/mL, progressive motility <32% and normal sperm morphology <4% with two times of semen analyses. 126 infertile men were recruited in the Department of Andrology, Urologic Medical Center, Shanghai General Hospital, Shanghai Jiao Tong University (Shanghai, China). 479 male infertile individuals were recruited in the Reproductive and Genetic Hospital of CITIC-Xiangya (Changsha, Hunan, China) and 120 in the Department of Reproductive Medicine, Xiamen Maternity and Child Care Hospital, Xiamen. (Fujian, Xiamen, China).

Patients with congenital diseases, deletions in the AZF gene, or factors linked to OAT, testicular cancer, cryptorchidism, or orchitis were excluded from the study. Patients who received chemotherapy or radiation were also excluded. The family histories of the patients were collected. For the control group, 20 healthy and fertility men from the Han Chinese ethnic group with a similar background of genetic makeup were included. Subjects of the control group exhibited normal semen analysis and they fathered one or more healthy babies.

Prior to participation, each participant signed a written informed consent form. The research was approved by the corresponding ethics committees of the Shanghai General Hospital, Shanghai Jiao Tong University(2021-SQ-112), the Reproductive and Genetic Hospital of CITIC Xiangya (LL-SC-2017-025 and LL-SC-2019-034), and the Xiamen Maternity and Child Care Hospital, Xiamen (Permit Number: 2020SQ199, KY-2019-060).

### Whole-exome sequencing

The manufacturer’s protocol was followed to extract whole DNA from blood samples of patients using TIANamp Blood DNA (TIANGEN Biotech, Beijing, China). Covaris focused ultrasonication was used for DNA fragmentation. xGen Exome Research Panel (Integrated DNA Technologies, Coralville, IA, USA) was used for capturing known exons and boundaries of the exon-intron followed by preparation of DNA sequences libraries according to the manufacturer’s protocol.

DNA was sequenced on a HiSeq X10 platform (Illumina, San Diego, CA, USA). Burrows-Wheeler Aliigner (BWA) was employed for the alignment of the sequencing reads with the human genome (GRCh37/hg19). GATK, VarSCan, Platypus, LoFreq, SNVer, Freebayes, VarDict and SAM tools were used for calling SNVs as well as indels within the intervals of captured coding exons. ANNOVAR software was used to filter and annotate variants. Those genetic variants were excluded which had higher than 1% allelic frequencies as per 1000 Gnomes Project and ExAC Browser while removing upstream, downstream and intronic variants. Frameshift, Nonsense, essential splice-site and missense variants with deleterious potential (SIFT, Mutation Taster and Polyphen-2) were retained for later analysis. Additionally, candidate genes were compared with pathogenic genes in mice (http://www.informatics.jax.org/mgihome/homepages/) and testis enriched genes in the database (http://www.proteinatlas.org/). The bioinformatic analysis and sequencing were performed with the collaboration of the Nuprobe company.

### Semen analysis, testicular biopsy for sperm extraction and ICSI treatment

Semen samples were collected from patients by asking them for masturbation, 3-7 days post-sexual intercourse. Analysis of the samples was performed in a laboratory as per 5^th^ WHO guidelines. The morphology of sperm was analyzed with examination using Diff-Quick staining. Ejaculated sperm of one patient (family 1, M1: II-1) without enough spermatozoa were recovered by testicular biopsy on the day of oocyte retrieval. H&E staining was performed on testicular tissue to assess spermatogenesis.

Viable sperm for ICSI were selected by laser, the tips of immotile sperm were targeted with a laser beam of approximately 200 μJ with an irradiation time of about 2 ms. Those spermatozoa which presented with curling of the tails after the laser shot were regarded as viable, while others which did not curling of the tails were considered to be non-viable. And assisted oocyte activation (AOA) has been done to activate oocytes. After ICSI treatment, outcomes were obtained from three different hospitals. Results of the following parameters were included: the number of injected oocytes, fertilized oocytes, ICSI cycles, rate of embryos cleavage, 8 cells, blastocysts, transfer cycles, per cycle transferred embryos, rate of implantation, pregnancies and miscarriage.

### Transmission electron microscopy analysis

Spermatozoa were fixed routinely as previously (5). After being embedded in Epon, ultrathin sections of sperm were stained with uranyl acetate, then photographed by TEM. At least 50 flagella with cross sections and several longitudinal sections were counted.

### ICSI derived from human ejaculated spermatozoa into mouse oocytes

To further prove the different capacity of ejaculated spermatozoa between patients with *TAF7L* variants and healthy men, microinjection of spermatozoa into the mice oocytes were performed using a previously described procedure (20). For superovulation, mice were injected with 5 IU of equine chorionic gonadotropin. Mice were then given 5 IU of human chorionic gonadotropin (HCG) after 48 hours. After 16 hours of HCG injection, cumulus-oocyte complexes were harvested from oviducts and transferred to HEPES Chatot-Ziomek-Bavister (CZB) medium. To disperse cells of cumulus, they were treated with hyaluronidase (0.1%). Oocytes were rinsed two times and then transferred to fresh CZB drop. They were given at least 15 min incubation in plain CZB media. To artificially activate the oocytes, they were kept for 1 hour in CZB free of Ca2+ and supplemented with SrCL2 (10mM) followed by washing two times again. For resuming incubation, they were again transferred to a CZB medium. 100 sperm from each group were microinjected into the eggs with piezoelectric elements. To examine the formation of pronucleus and *in vitro* development, the oocytes in the CZB medium were kept for incubation at 37 °C and 5% CO2.

### Immunofluorescence staining

The spermatozoa of the proband (family 1, M1: II-1) and normal control were stained by immunofluorescent. The samples were fixed with 4% paraformaldehyde for 20 min. After 10 min washes with PBS, heat-induced antigen retrieval was carried out by boiling the slides in 10 mM citrate buffer (pH 6.0) with a microwave oven for 10 min. After three 10 min washes with 0.1% Triton X-100 in PBS, the slides were blocked with 5% BSA diluted in PBST for 1h and then the slides were incubated with anti-histone H3 (1:100; CST), anti-PRM2(1:100; Atlas) in PBST overnight, samples were incubated with fluorescein isothiocyanate-conjugated anti-rabbit IgG (1:200; Invitrogen), and peanut agglutinin (PNA) (1:300; Invitrogen) for 30 min at room temperature. The samples were washed three times in PBS and incubated 10-min with Hoechst33342(1:200; Invitrogen), imaged by fluorescence microscopy.

### RNA extraction and reverse transcriptase polymerase chain reaction

According to the manufacturer’s instruction, testicular RNA of patient (Family 1, M1: II-1) was extracted using the kit (Qiagen, USA). The purity was assessed by the A260/A280 ratio. Then we carried out the reverse transcription under the following conditions: 37 °C for 15 min, followed by 85 °C for 5 s. The products were performed under the following conditions: 95 °C for 30s, 40 cycles of 95 °C for 10 s, 60 °C for 30 s, and 95 °C 15s, 60°C for 60s, 95°C for 15s. GAPDH was used as an internal control and primers for real-time quantitative PCR are listed in **Table S1**.

### Expression plasmid construction and transfection

Full-length cDNA encoding human *TAF7L* was amplified and cloned into pCMV7.1 vector with N-terminal FLAG tag. Site-directed mutagenesis was accomplished using a ClonExpress Ultra One Step Cloning Kit (C115-01, Vazyme, China). The wild-type and mutant clones were confirmed by Sanger sequencing. HEK293T cells were cultured in DMEM/high glucose (SH30243.FS, Cytiva) with 10% FBS (10099141C, Gibco) and 1% penicillin/streptomycin (15140122, Gibco) at 37°C in 5% CO2. *TAF7L* wild-type and mutant plasmids were transfected into HEK293T cells using the lipofectamine 3000 (L3000015, Invitrogen) according to the manufacturer’s protocol.

### Western blot

48 h after transfection, the cells were lysed by RIPA lysis buffer (89901, Thermo Fisher Scientific) on ice 20 min, followed by centrifugation at 14,000 × g for 15 min at 4°C to isolate the protein, which was quantified by BCA assay (23225, Thermo Fisher Scientific) and separated by 10% sodium dodecyl sulfate polyacrylamide gels and were transferred to a 0.22 µm PVDF membrane (ISEQ00010, Millipore). The membranes were blocked with 5% not fat milk in TBST buffer 1h. After washing three times with TBST for 5 min, then incubated at 4°C overnight with anti-FLAG rabbit monoclonal antibodies (dilution: 1:5,000; catalogue number: F1804, Sigma) or β-actin (dilution: 1:10,000; 66009-1-Ig, Proteintech). The membranes were washed with TBST three times and incubated with anti-Mouse IgG HRP (dilution: 1:5,000; sc-516102, Santa Cruz Biotechnology) for 1 h at room temperature followed by washing again with TBST three times. Finally, the blots were imaged on AI600 imager (GE healthcare)

## Results

### Clinical characteristics

In this study, WES of 725 idiopathic OAT patients was performed. Four X-linked hemizygous deleterious variants of *TAF7L* were identified in five probands (0.68%,5/725).

Patients were examined for physical parameters and they exhibited normal epididymis, scrotum, prostate, penis and testes development. Semen analysis revealed OAT phenotype as per the 5^th^ edition of WHO guidelines. All patients had normal sex hormones, sex chromosomes and autosomal chromosomes. No Y chromosome microdeletions and no history of cancer, cryptorchidism, hypogonadism, alcoholism, or smoking were found.

### Identification of hemizygous *TAF7L* variants in men with OAT

**Figure 1:**
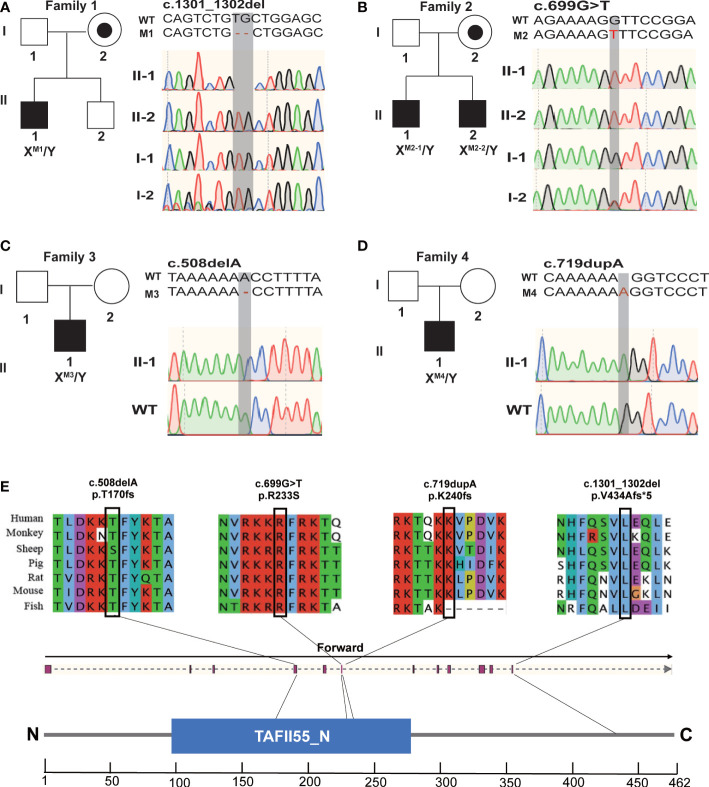
Hemizygous mutations in *TAF7L* identified in patients with OAT. **(A–D)** Pedigree analysis of the four patients affected by X-linked *TAF7L* Hemizygous variants that were identified by WES. Black filled squares indicate infertile men in these families. Sanger sequencing results are shown to the right of the pedigrees. The mutated positions are indicated by red words and black shadow boxes. **(E)** The genomic structure of *TAF7L*, with variants mapped to isoform 1 (GenBank accession number, NM_024885) The positions of the novel *TAF7L* variants identified in this study are indicated by black lines. Locations of the mutated sites in the exon structure of *TAF7L*(upper); Locations of the affected amino acids in the protein domain map of TAF7L (bottom). Sequence alignment shows conservation of the mutated residues among different species. Blue squares stand for TAFII55 protein conserved region according to the NCBI.

To identify candidate genes that could be potentially associated with OAT, WES was performed. The workflow of data processing and analyses was described in **Supplementary Figure 1**. After excluding the polymorphisms, which were reported in previous studies. We found four deleterious variants X-Linked *TAF7L* variants in five Chinese OAT patients, the hemizygous frameshift [c.1301_1302del;(p.V434Afs*5)] in M1(**Figure 1A**; **Table 1**), a hemizygous missense mutation [c.699G>T;(p.R233S)] in M2-1 and M2-2, which was inherited from their parents (**Figure 1B**; **Table 1**). We also found the hemizygous frameshift [c.508delA; (p. T170fs)] in M3, the hemizygous frameshift [c.719dupA;(p.K240fs)] in M4 (**Figures 1C, D**; **Table 1**).

**Table 1:** Hemizygous deleterious *TAF7L* variants induce OAT in Chinese men.

*TAF7L* variants	M1	M2-1/M2-2	M3	M4
cDNA alteration	c.1301_1302del	c.699G>T	c.508delA	c.719dupA
Variant allele	Hemizygous	Hemizygous	Hemizygous	Hemizygous
Protein alteration	p. V434Afs*5	p. R233S	p. T170fs	p. K240fs
Variant type	Frameshift	Missense	Frameshift	Frameshift
Allele frequency in human population				
1000 Genomes Project	0	0	0	0
East Asians in gnomAD	0	0	0	0
All individuals in gnomAD	0	0	0	0
Function prediction				
Mutation Taster	Damaging	Damaging	Damaging	Damaging
SIFT	N/A	Damaging	N/A	N/A
PolyPhen-2	N/A	Damaging	N/A	N/A

NCBI reference sequence number of TAF7L in GenBank: NM_024885. N/A, not available.

*means stop.

These hemizygous *TAF7L* variants were not detected in human population genome databases, including gnomAD and the 1000 Genomes Project (**Table 1**). *In silico* analysis of all these variants predicted them to be deleterious (SIFT, PolyPhen-2, M-CAP and CADD tools) (**Table 1**). Hence, these variants were classified as pathogenic following the American College of Medical Genetics and Genomics criteria (ACMG). All corresponding variant residues are highly conserved in many organisms (**Figure 1E**).

### ICSI treatment may rescue the damaged male fertility carrying *TAF7L* mutation with sperm of testes

It is suggested that ICSI treatment can be effective in overcoming the physical problems faced by sperm from patients. The couples underwent ICSI by using spermatozoa of the subjects carrying hemizygous *TAF7L* mutation. Hence, the patients with *TAF7L* variants got fewer two-cell embryos and blastocysts than the control. These five patients with *TAF7L* variants didn’t get clinical pregnancy using ejaculated sperm, two of them chose donor’s sperm for ICSI treatment.

One patient (Family 1, M1: II-1), who had previously undergone seven ICSI cycles without conceiving with ejaculated sperm. In this time, ejaculated sperm of patient without enough spermatozoa were recovered by testicular biopsy on the day of oocyte retrieval. The couple got the successful clinical pregnancy with sperm of testes at the time of writing (**Table 2**).

**Table 2:** Clinical outcomes of ICSI cycles using the spermatozoa from men harboring hemizygous *TAF7L* variants.

Subject	M1	M2-1	M2-2	M3	M4	
Male age (years)	32	34	27	33	35	
Female age (years)	29	31	-	32	-	
Number of ICSI cycles	3	4	N/A	2	N/A	
Number of oocytes injected	15	21	-	13	-	
Number (and rate) of fertilized oocytes	6 (40%)	4 (19%)	–	5 (38%)	–	
Number (and rate) of cleavage embryos	2 (33%)	1 (25%)	-	0	-	
Number (and rate) of 8-cells	N/A	N/A	–	–	–	
Number (and rate) of blastocysts	N/A	N/A	-	-	-	
Number of transfer cycles	N/A	N/A	-	-	-	
Number of embryos transferred per cycle	1	1	-	-	-	
Implantation rate	1	1	-	-	-	
Clinical pregnancy rate	1	0	-	0	-	
Miscarriage rate	1	-	-	-	-	

−, not applicable; N/A, not available

**Figure 2:**
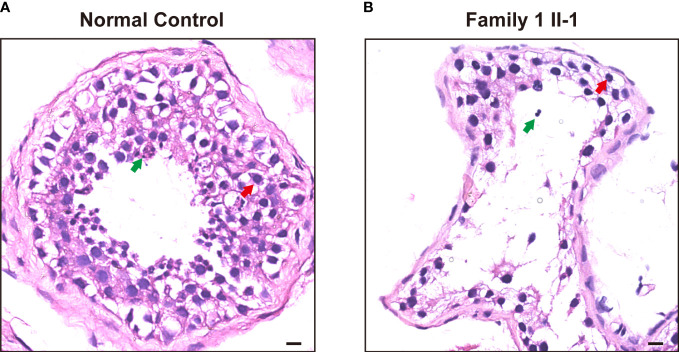
H&E staining of cross-sections in the subjects carrying hemizygous mutations in *TAF7L* and a patient with OA. **(A)** H&E staining of cross-sections of testicular biopsy from a patient with OA as the positive control and **(B)** the Family 1 II-1patient. Red arrows mark the spermatocyte. Green arrows mark the spermatozoa. Scale bars: 10μm.

As contrast normal spermatogenesis, histological analysis of testis revealed that this patient (Family 1, M1: II-1) showed post meiotic arrest at the first stage of spermiogenesis, which revealed dramatically decreased elongated spermatids (**Figure 2**).

### Reduced *in-vitro* fertilization capacity from patients with *TAF7L* variants

**Figure 3:**
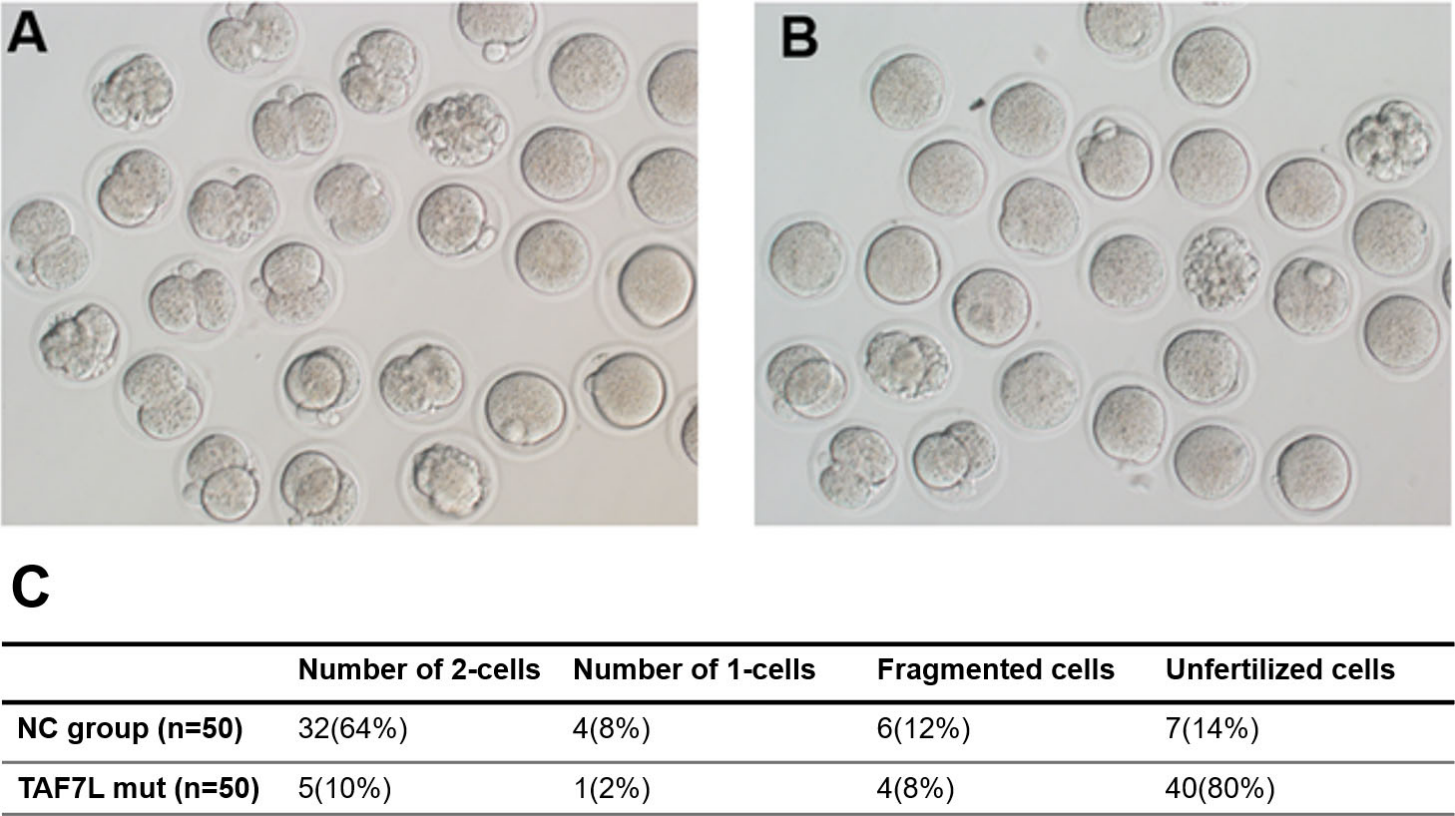
Embryos developmental potentials from oocytes of mouse fertilized with sperm carrying hemizygous mutations in *TAF7L* and the healthy man. **(A)** Two cells embryo formation from the ejaculated of healthy man. **(B)** Two cells embryo formation from ejaculated of the patient (Family 1 II-1) presented with OAT phenotype. **(C)** Statistics of pregnancy outcome from the healthy man and patient. NC: normal control.

To ascertain the fertilization capacity of sperm from patients with *TAF7L* variants, microinjection of spermatozoa into the mice oocytes were performed to demonstrate the capacity of spermatozoa derived from OAT-affected patients with *TAF7L* variants. Notably, the embryos from both *TAF7L* variants and control could develop into the two-cell stage. However, the two-cell rate was significantly reduced compared with the control (10% *vs* 64%) (**Figure 3**). Collectively, it is illustrated that the sperm from OAT affected patients with *TAF7L* variants showed reduced fertilization capacity compared to the normal sperm.

### Defective chromatin compaction in men carrying hemizygous *TAF7L* variants

**Figure 4:**
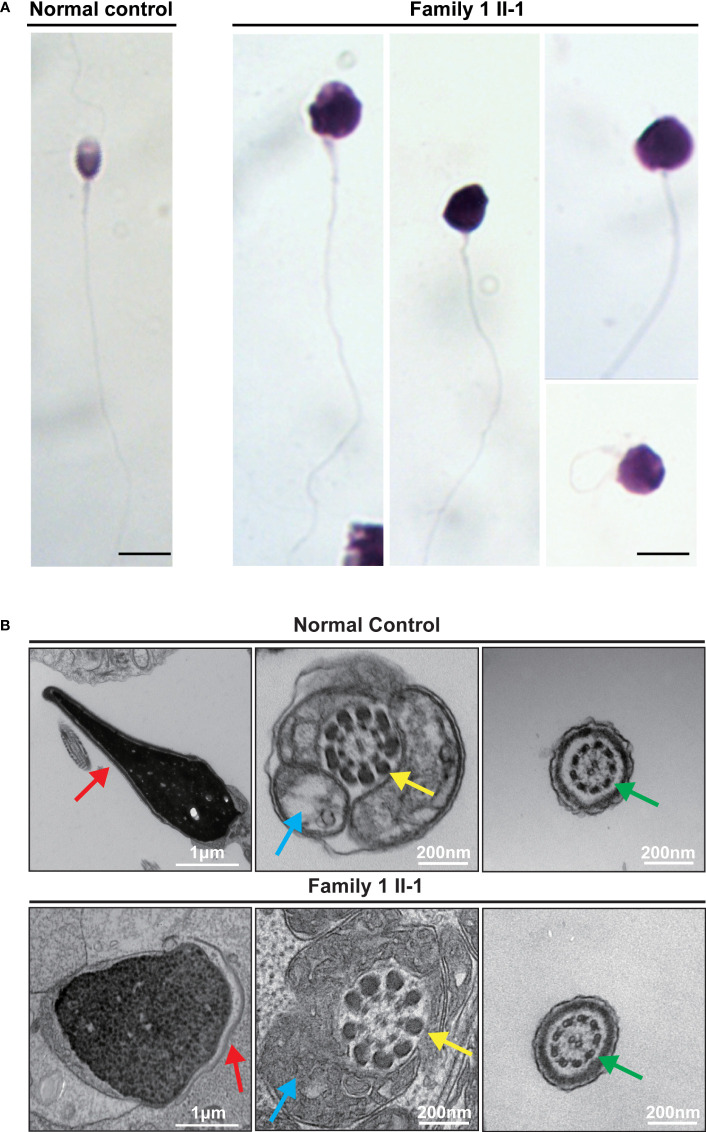
Sperm morphology and Ultrastructure Analysesin for the subjects carrying hemizygous mutations in *TAF7L.* **(A)** The morphology of the spermatozoa from a fertile control individual and subject Family 1 II-1 under light microscopy. Multiple images were taken, and typical features of abnormal spermatozoa are exemplified, such as abnormal head. Scale bars: 10 μm. **(B)** The longitudinal sections of sperm flagellar of a control individual. The normal sperm had a symmetrical mid-piece with smooth axoneme surrounding with a regularly arranged mitochondrial sheath. Family 1 II-1 sperm showed the less condensed head. Red arrows mark the acrosome. Blue arrows mark the mitochondrial sheath. Green arrows mark the axoneme. Yellow arrows mark the outer dense fibers.

The parameters for semen analysis of *TAF7L* variants were obtained from laboratories according to the WHO^5th^ guidelines. Semen analysis indicated severe reduced sperm concentration in all of five men harboring hemizygous *TAF7L* variants (**Table 3**). Furthermore, Diff-Quik staining displayed sperm head deformity (**Figure 4A**). Moreover, their nuclei were more efficiently stained with acidic aniline (**Figure 4A**), implying less condensed chromatin in these sperm head. TEM verified that the heads of sperm were less condensed in comparison with the controls (**Figure 4B**). These observations indicated of defective chromatin compaction in the heads of sperm.

**Table 3:** Semen characteristics and sperm morphology in men harboring hemizygous *TAF7L* variants.

Subject	M1	M2-1	M2-2	M3	M4	Reference Limits
Semen parameter						
Semen volume (mL)	2.0	2.0	1.5	1.5	1.5	1.5^a^
Sperm concentration (10^6^/mL)	<1.0	<1.0	<1.0	<1.0	<1.0	15.0^a^
Motility (%)	0	0	0	0	0	40.0^a^
Progressive motility (%)	0	0	0	0	0	32^a^
Sperm morphology						
Head defects (%)	95	92	94	97	89	>4^b^
Neck and midpiece defects (%)	5	8	6	3	11	
Tail defects (%)	0	0	0	0	0	
Excess residual cytoplasm (%)	0	0	0	0	0	
Irregular caliber (%)	100	100	100	100	100	

N/A, not available.

a Reference limits according to the WHO standards.

b Reference limits according to the WHO standards: Normal sperm morphology rate

### Abnormal histone-to-protamine during spermiogenesis

**Figure 5:**
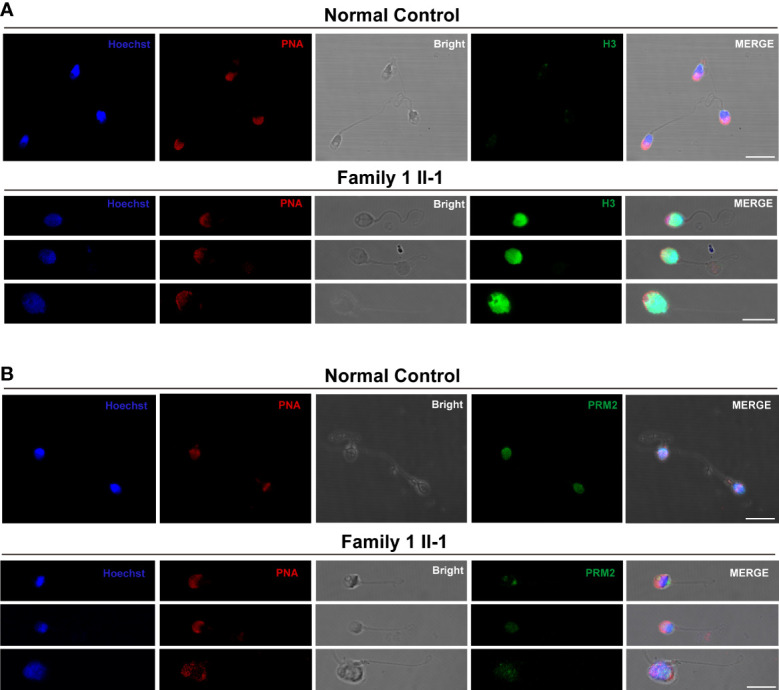
The Histone-to-Protamine Exchange Is Impaired during Spermiogenesis in the subjects carrying hemizygous mutations in *TAF7L.* **(A)** Representative confocal images of immunostaining with Hoechst (blue), PNA (red), Bright, and anti-H3 antibody (green) on sperm from *TAF7L* variants -affected patients and normal man. Scale bars: 10μm. **(B)** Representative confocal images of immunostaining with Hoechst (blue), PNA (red), Bright, and anti-PRM2 antibody (green) on sperm from *TAF7L* variants -affected patients and normal man. Scale bars: 10μm.

To figure out the potential causes underlying defective chromatin compaction in the heads of sperm. Immunofluorescence staining verified abnormal retention of histone H3, and conversely, drastic reduced levels of protamine PRM2 in the heads of sperm (Family 1, M1: II-1) contrast to control (**Figure 5**).

**Figure 6:**
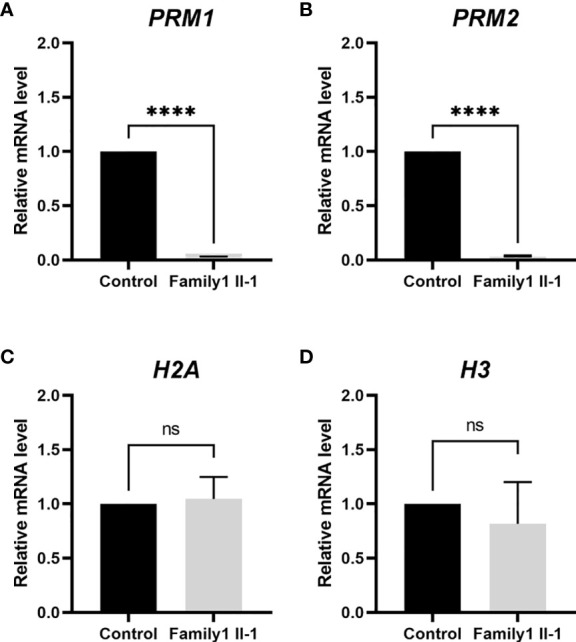
Differential abundance of the *PRM1*, *PRM2*, *H2A* and *H3* transcripts in the subject carrying hemizygous mutations in *TAF7L* and normal men. **(A–D)** The mRNA content of *PRM1* and *PRM2* is significantly lower in *TAF7L* variants -affected patient compared with normal man. *H2A* and *H3* mRNA ratio in *TAF7L* variants -affected patient samples are close to normal man. NS, not significant; **** indicates p <0.0001.

Subsequently, As shown in **Figure 6**, RT-PCR analysis revealed that the testicular tissue of patient (Family 1, M1: II-1) had significantly reduced expression of protamine *PRM2* mRNA (p < 0.001). And these experiments indicated impaired histone-to-protamine exchange during spermiogenesis.

### *In vitro* protein expression of *TAF7L* variants

**Figure 7:**
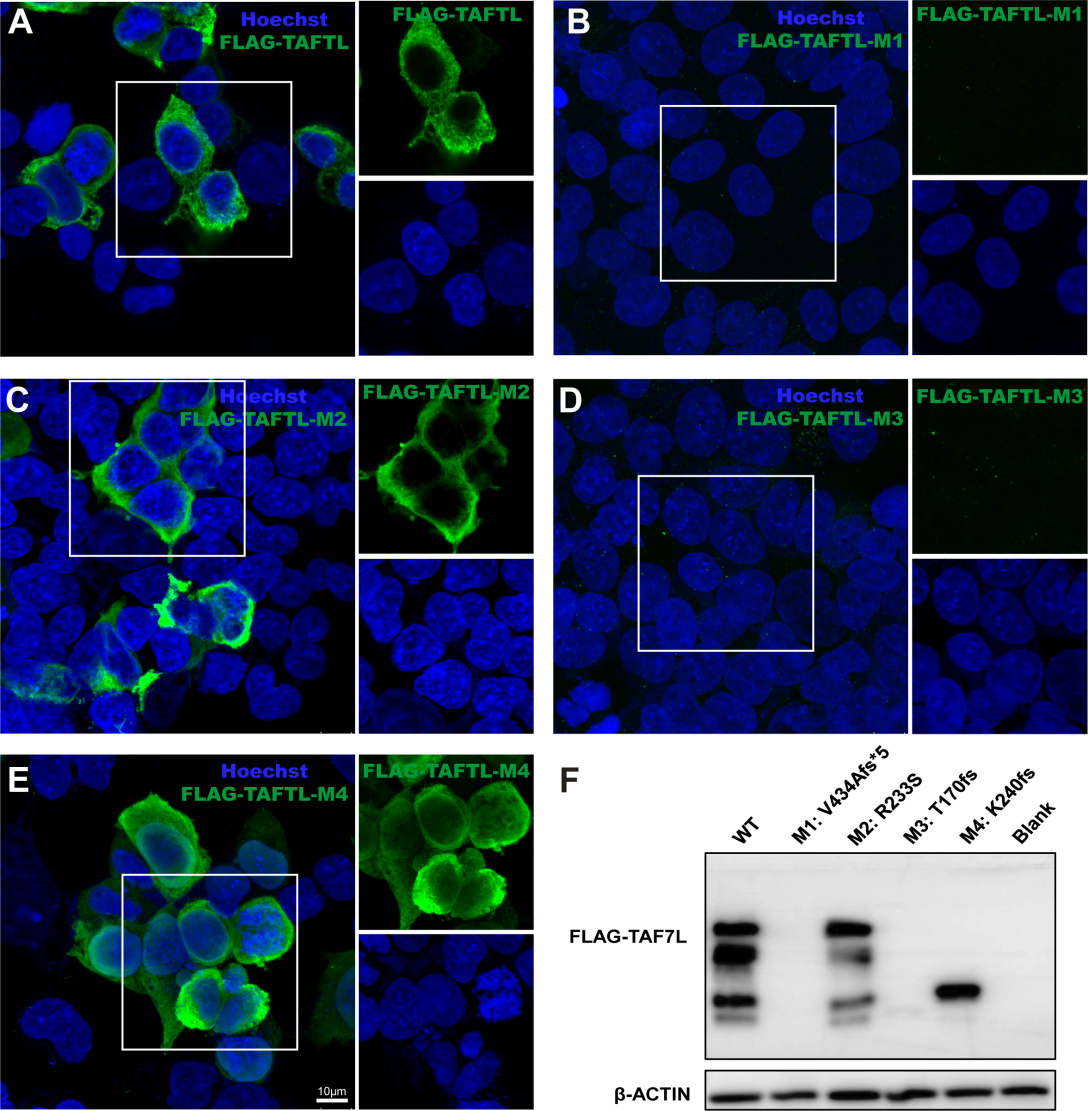
Effects of the *TAF7L* variants in *in vitro*. **(A–E)** Immunofluorescence analysis of TAF7L expression in HEK293T cell transfected with FLAG-tagged wild-type and mutant plasmids. **(F)** Western blot analysis of TAF7L-3×FLAG protein level in HEK293T transfected with wild-type and mutant plasmids.

To determine the functional phenotypes of missense and LoF variants of human *TAF7L* variants *in vitro*. 293T cells were transiently transfected with the *TAF7L* wild-type and four mutant *TAF7L* plasmids with 3×FLAG tagged. Compared to wild-type, Immunofluorescence analysis showed that variant c.1301_1302del (p. V434Afs*5) and c.508delA (p. T170fs) had no immunofluorescence signal was detected above the background (**Figures 7A, B, D**), c. 719dupA (p. K240fs) signal was localized massively within the nucleus (**Figure 7E**). But variant c.699G>T;(p. R233S) location were no different compared to WT (**Figure 7C**). The WB data were similar with the IF results. The c. 1301_1302del (p. V434Afs*5), c. 508delA (p. T170fs) and c.719dupA (p. K240fs) mutations yielded a truncated protein, c. 1301_1302del (p. V434Afs*5) and c.508delA (p. T170fs) showed nearly undetectable expression protein level and that the c.719dupA (p. K240fs) mutation generated a normal expression truncated protein. There was no change in protein level for the c. 699G>T (p. R233S) mutation (**Figure 7F**).

## Discussion

As discussed above, we found hemizygous mutations of *TAF7L* among five non-related OAT patients (0.68%,5/725). The samples were collected from three different hospitals. The WES results showed that the subjects did not carry any other known pathogenic variant related to OAT genes except the hemizygous *TAF7L* mutation. Remarkably, all the *TAF7L* mutations were either completely missing in the public databases (1000 Genomes and gnomAD) or they showed low allelic frequencies. They were indicated to be potentially pathogenic by *in-silico* study. Therefore, it is needed to explore the OAT phenotypes in such cases by hemizygous deleterious variants in *TAF7L.*

*TAF7L* (also known as CT40) is a paralogue of *TAF7* which is a subunit of TFIID and is specific to germ cells. *TAF7L* encodes a protein associated with transcription factors and is mainly expressed in testis (21). Previously it was found that mice deficient with *TAF7L* exhibited low fertility, abnormal morphology of sperm and reduced motility (22).Previous study mutation frequencies in *Taf7l* gene were likely to be polymorphisms in human. Only one study previously reported a deleterious mutation (D136G) in the X-linked *TAF7L* gene as a potential cause of oligozoospermia in men, but the morphology of sperm was not described in this study. And the corresponding D144G substitution in the mouse *Taf7l* gene does not affect male fertility (23).Therefore, this is a great clinical heterogeneity in man with *Taf7l* variants.

Herein, we identified four deleterious variants *TAF7L* in human. Instead of causing oligozoospermia, the mutation causes OAT, including a hemizygous frameshift mutation of *TAF7L* [c. 1301_1302del;(p. V434Afs*5)] in M1(**Figure 1A**), one hemizygous missense mutation [c. 699G>T;(p. R233S)] in M2-1 and M2-2, it was confirmed that this variant was inherited from their parents (**Figure 1B**). We also found the hemizygous frameshift mutation [c. 508delA;(p. T170fs), c. 719dupA;(p. K240fs)] in M3 and M4, respectively, (**Figures 1C, D**; **Table 1**). These four *TAF7L* variants were not found in the control group of human population genome databases and *in silico* analysis predicted as deleterious. *In vitro* tests confirmed the protein-truncating mutation c. 1301_1302del;(p. V434Afs*5), c. 508delA;(p. T170fs), c. 719dupA;(p. K240fs) causes abnormal expression of TAF7L.

No medicine is available for the treatment of OAT patients which can improve the quality of the ejaculated spermatozoa, which renders ICSI as the only solution for conception. However, studies have found that different mutations in various genes cause OAT with various types of clinical outcomes. For instance, biallelic mutations of CEP135 (MIM: 301057) resulted in pregnancy failure, while oligozoospermia due to CFAP47 (MIM:301057) mutation showed good clinical results (12). In this study, the outcomes of the ICSI treatment from three different hospitals showed that the number of blastocysts and two cells embryo formation by *TAF7L* variants was lower compared to the control.

In the current study, these three patients with *TAF7L* variants didn’t get clinical pregnancy using ejaculated sperm, two of them chose donor’s sperm for ICSI treatment. Ejaculated sperm of patient (Family 1, M1: II-1) without enough spermatozoa were recovered by testicular biopsy on the day of oocyte retrieval. The couple got successful clinical pregnancy at the time of writing (**Table 2**). Based on the current clinical evaluation of female fertility, we believe that their fertility were normal. Hence, this study supports the idea that male infertility could be because of hemizygous *TAF7L* variants due to the low fertilization capacity of sperm.

To further prove this hypothesis that the capacity of low spermatozoa derived from OAT patients with *TAF7L* variants, microinjection of spermatozoa into the mice oocytes was performed. The two-cell rate was significantly reduced than the control (10% *vs* 64%). This indicated the sperm from OAT patients with *TAF7L* variants showed reduced *in-vitro* fertilization capacity through ICSI treatment.

Why the low capacity of spermatozoa derived from OAT patients with *TAF7L* variants? In this study, we confirmed that the defective chromatin compaction in sperm head of proband (Family 1, M1: II-1). Previous study indicated that Taf7l and Trf2 coregulate the expression of protamine 1/2 (*Prm1/2*) genes, and ChIP-qPCR analysis confirmed dramatically diminished in Taf7l-null testes at target protamine 1/2 (*Prm1/2*) promoters (24). In this study, we found that abnormal retention of histone *H3* and reduced levels of protamine *PRM2* were in the heads of sperm. RT-PCR analysis revealed that the testicular tissue of patient (Family 1, M1: II-1) had significantly reduced expression of protamine *PRM2* mRNA, So we speculated that the abnormal of histone-to-protamine exchange may induce the defective chromatin compaction of sperm head. Because the histone-to-protamine exchange is crucial for producing functional sperm, these results implied that a defect histone-to-protamine exchange may underlie the capacity deficiency of *TAF7L* variants sperm.

## Data availability statement

The datasets for this article are not publicly available due to concerns regarding participant/patient anonymity. Requests to access the datasets should be directed to the corresponding authors.

## Ethics statement

The research was approved by the corresponding ethics committees of the Shanghai General Hospital, Shanghai Jiao Tong University(2021-SQ-112), the Reproductive and Genetic Hospital of CITIC Xiangya (LL-SC-2017-025 and LL-SC-2019-034), and the Xiamen Maternity and Child Care Hospital, Xiamen (Permit Number: 2020SQ199, KY-2019-060)

## Author contributions

CY, ZL and EZ designed the study, directed and supervised the research. HB, YS and YT collected the data. YZ, JX, SX, performed experiments. PL and ZJ have drafted the work. XW, WC, JZ enrolled the patients and collected clinical information. HB wrote the manuscript, with input from others. CY, ZL and EZ substantively revised it. All authors contributed to the article and approved the submitted version.
