# Supplementary material for: Deleterious variants in TAF7L cause human oligoasthenoteratozoospermia and its impairing histone to protamine exchange inducing reduced in vitro fertilization
Source: Front Endocrinol (Lausanne). 2023 Jan 11;13:1099270. doi: 10.3389/fendo.2022.1099270 (PMC9874084; doi:10.3389/fendo.2022.1099270)
Supplement: Supplementary file 1 [file DataSheet_1.docx]

| **TableS1. Primers Used for RT-qPCR and RT-PCR Assays** | | |
| --- | --- | --- |
| **Primer Names** | **Primer Sequences (5'-3')** | **Tm** |
| H-H2A-F | TTTGTTGCGAGGTTCTGAGC | 59°C |
| H-H2A-R | CTTGTTTGCCACGACCAGAC |  |
| H-H3-F | TTAAGACGGACCTGCGCTTC | 60°C |
| H-H3-R | CTTGGGCATAATGGTCACGC |  |
| H-PRM1-F | CCGGAGCAGATATTACCGCC | 59°C |
| H-PRM1-R | CTACATCGCGGTCTGTACCT |  |
| H-PRM2-F | ATCGCAGAGGCTGCAGAAC | 60°C |
| H-PRM2-R | TTGGTGTTTCGGGCGACTTT |  |
