# Supplementary figures and images for: Deleterious variants in TAF7L cause human oligoasthenoteratozoospermia and its impairing histone to protamine exchange inducing reduced in vitro fertilization
Source: Front Endocrinol (Lausanne). 2023 Jan 11;13:1099270. doi: 10.3389/fendo.2022.1099270 (PMC9874084; doi:10.3389/fendo.2022.1099270)

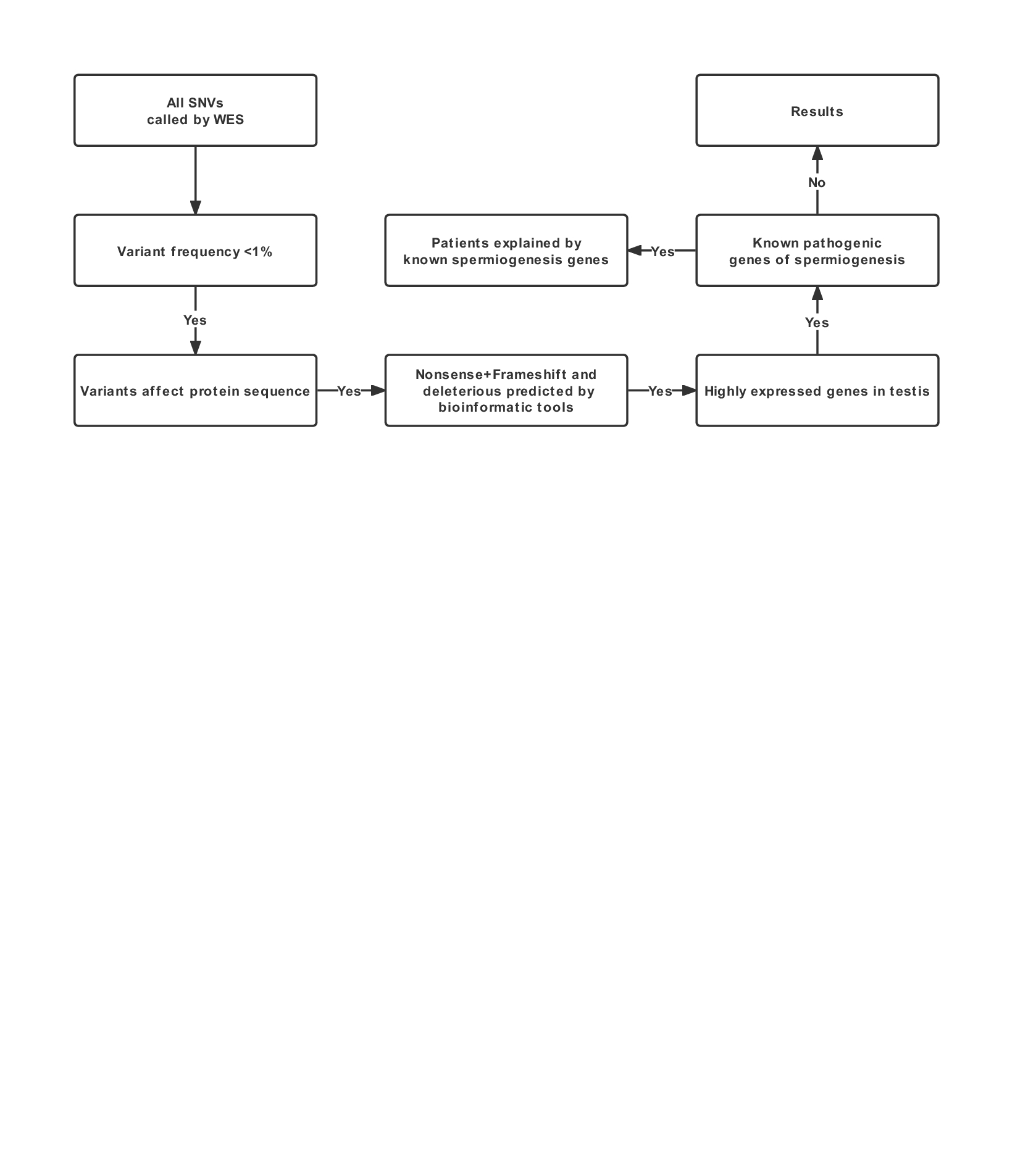

Supplement: Supplementary file 2 [file Image_1.tif]
